# Developmental programming of hypothalamic melanocortin circuits

**DOI:** 10.1038/s12276-021-00625-8

**Published:** 2022-04-26

**Authors:** Sebastien G. Bouret

**Affiliations:** 1 Inserm, Laboratory of Development and Plasticity of the Neuroendocrine Brain, Lille Neuroscience & Cognition Research Center, UMR-S 1172, Lille, 59000 France; 2 University of Lille, FHU 1,000 Days for Health, Lille, 59000 France

**Keywords:** Hypothalamus, Obesity

## Abstract

The melanocortin system plays a critical role in the central regulation of food intake and energy balance. This system consists of neurons producing pro-opiomelanocortin (POMC), melanocortin receptors (MC4Rs), and the endogenous antagonist agouti-related peptide (AgRP). *Pomc* and *Mc4r* deficiency in rodents and humans causes early onset of obesity, whereas a loss of *Agrp* function is associated with leanness. Accumulating evidence shows that many chronic diseases, including obesity, might originate during early life. The melanocortin system develops during a relatively long period beginning during embryonic life with the birth of POMC and AgRP neurons and continuing postnatally with the assembly of their neuronal circuitry. The development of the melanocortin system requires the tight temporal regulation of molecular factors, such as transcription factors and axon guidance molecules, and cellular mechanisms, such as autophagy. It also involves a complex interplay of endocrine and nutritional factors. The disruption of one or more of these developmental factors can lead to abnormal maturation and function of the melanocortin system and has profound metabolic consequences later in life.

## Brain hormones: The melanocortin system in health and disease

The melanocortin system of the brain regulates feeding behavior, energy balance and body weight, and investigating its development is helping researchers understand and explore treatments for chronic diseases, including obesity and type-2 diabetes. Sebastien Bouret at the University of Lille, France, reviews research into the role of the melanocortin system’s development in health and disease. The system consists of nerve cells that produce the precursor of peptide hormones called pro-opiomelanocortins, protein receptor molecules that interact with melanocortins, and peptide molecules that counteract and regulate melanocortin effects. The review discusses the major steps in the developmental programming of the melanocortin system and the hormonal, molecular, cellular, and nutritional factors influencing the nerve cells involved. Some chronic diseases may originate from disruption of the melanocortin system’s development in the embryo and soon after birth.

## Introduction

The melanocortin system is a critical component of brain pathways that regulate feeding behavior and energy homeostasis. The brain’s melanocortin system consists of pro-opiomelanocortin (POMC) neurons and neurons that produce agouti-related peptide (AgRP), an endogenous inverse agonist of melanocortins. There are two anatomically distinct POMC neuronal populations in the brain. The largest population of POMC neurons resides in the arcuate nucleus of the hypothalamus (ARH). A discrete population of POMC neurons is also found in the nucleus of the solitary tract (NTS) in the brainstem. In contrast, AgRP neurons are exclusively located in the ARH and coexpress neuropeptide Y (NPY). Pharmacological approaches revealed more than three decades ago that melanocortins have a potent and long-lasting inhibitory effect on feeding^1^. More recently, neuron- and gene-specific genetic studies have shown the importance of POMC neurons in mediating the physiological actions of metabolic hormones, such as leptin and insulin^2,3^. Opto- and chemogenetic experiments further indicated that although arcuate POMC neurons appear to be more involved in integrating long-term adiposity signals, the contribution of hindbrain POMC neurons seems to be more specific to the integration of short-term satiety signals^4,5^. In contrast, optogenetic stimulation of AgRP neurons rapidly evokes feeding^4^, whereas genetic ablation of AgRP neurons in adult animals causes starvation^6,7^.

The POMC-derived peptide alpha-melanocyte-stimulating hormone (αMSH) and AgRP modulate the activity of the melanocortin 4 receptor (MC4R). αMSH activates MC4R, while AgRP acts as an endogenous inverse agonist of MC4R, meaning that it suppresses constitutive MC4R activity and simultaneously antagonizes the effects of αMSH. The highest expression of MC4Rs is found in the hypothalamus and the brainstem^8–10^. Target neurons expressing MC4Rs include neurons producing oxytocin, thyrotropin-releasing hormone, and corticotrophin-releasing hormone in the paraventricular nucleus of the hypothalamus (PVH)^10,11^. The melanocortin system is universal and common to all mammals, including humans, nonhuman primates, and rodents, and mutations in the MC4R gene are the most common monogenic disorders that cause obesity in humans^12^.

In this review, we will first describe the major steps of melanocortin system development. We will also discuss the hormonal, molecular, cellular, and nutritional factors influencing the development of POMC and AgRP neurons.

## Ontogenesis of the hypothalamic melanocortin system

**Fig. 1:**
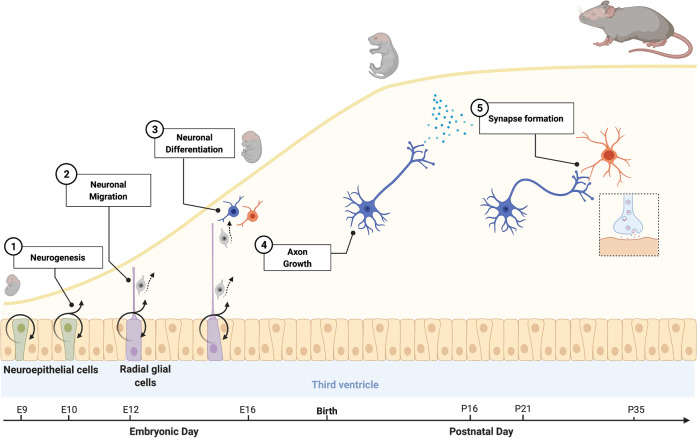
Important periods of hypothalamic development. The development of a functional hypothalamus occurs in two major phases: the determination of cell numbers, which includes neurogenesis, neuronal migration, and cell fate, and the formation of functional circuits, which includes axon growth and the formation of functional synapses. In rodents, neurogenesis, neuronal migration, and cell fate occur during mid-to-late gestation, while axon growth and synapse formation occur primarily postnatally.

POMC and AgRP neurons are derived from precursor cells found in the proliferative zone located in the inner and lower portions of the third ventricle, also known as the neuroepithelium of the third ventricle^13^ (Fig. 1). The most comprehensive animal models used to study the development of the melanocortin system are rodent models (mainly mice and rats), and a limited number of studies have also examined the development of this neuronal system in humans and nonhuman primates. Birth dating approaches using the thymidine analog bromodeoxyuridine 5-bromo-2′-deoxyuridine (BrdU) indicated that the majority of POMC and AgRP/NPY neurons in the mouse ARH are born primarily on embryonic day (E)11–E12^14,15^ (Fig. 1). However, some POMC neurons, which are located more laterally in the ARH, are generated as late as E13. Gene expression studies showed that neurons in the presumptive ARH begin to express *Pomc* mRNA on E10–E12, whereas *Npy* mRNA expression is not observed until E14 (ref. ^15^). These observations are consistent with the early determination of *Pomc* cell fate. Intriguingly, genetic cell lineage tracing studies revealed that approximately half of the *Pomc*-expressing precursors acquire a non-POMC fate in adult mice, and nearly one-quarter of the mature NPY neurons in the ARH share a common progenitor with POMC cells^15^. These data show the unique property of *Pomc*-expressing progenitors with respect to giving rise to antagonistic neuronal populations. Differentiated neurons then send out axonal processes to other target nuclei to convey neuronal information and control behavior. The majority of arcuate POMC and AgRP neurons have relatively short axons and connect primarily to neurons within the hypothalamus^16^. Axonal projections from the arcuate nucleus develop postnatally and provide inputs to the PVH between postnatal day (P) 8 and P10 in mice^16^ (Fig. 1). Using immunohistochemical techniques, Grove et al.^17^ confirmed that projections immunopositive for AgRP are immature at birth and develop mainly during the second week of postnatal life in rats. The same temporal pattern was observed for the development of POMC projections in mice^18^.

*Mc4r* mRNA is first expressed at E12 in the proliferative zone surrounding the lower portion of the third ventricle (also known as the neuroepithelium), and its expression peaks at E16 (ref. ^19^). These findings are particularly interesting because, as described above, it is known that neurons that compose various hypothalamic nuclei in adults are primarily derived from precursors that originate from this proliferative zone, raising the possibility that MC4R could be involved in hypothalamic neurogenesis. However, further studies are needed to determine the developmental stage during which MC4R becomes functional. Nevertheless, the observation that peripheral injection of the melanocortin agonist melanotan II (MTII) reduces milk intake and body weight as early as during the first 2 weeks of postnatal life suggests that MC4R receptors are present and functional in the hypothalamus at least soon after birth^20^. Consistent with this idea, in situ hybridization analysis showed that *Mc4r* mRNA is abundant in the hypothalamus, especially in the PVH at P10. Peripheral injection of MTII induces strong induction of cFos immunoreactivity (a marker of neuronal activation) in the PVH at P5–P15, further supporting the functionality of MC4R in the PVH during early postnatal life^20^. Moreover, Melnick et al.^21^ showed an age-dependent increase in the electrophysiological response of specific sets of PVH neurons to melanocortins, with a maximal response observed at P28–P35, suggesting that synapses between POMC and AgRP axons and PVH MC4R^+^ target neurons continue to build until puberty (Fig. 1).

## Consideration of species differences in melanocortin circuit development

The marked differences in the normal ontogeny of hypothalamic development between rodents and human and nonhuman primates warrant attention. First, the regional development of the rodent hypothalamus proceeds on a timeline of days in rodents versus weeks to months in human and nonhuman primates. Second, although rodents exhibit considerable postnatal hypothalamic development, human and nonhuman primates undergo considerably more prenatal maturation of hypothalamic circuits. For example, although the hypothalamus is not mature until after weaning in rodents, hypothalamic neurogenesis and axon growth occur primarily during intrauterine life in nonhuman primates, including humans. Hypothalamic development has been studied in detail by Grove and collaborators in Japanese macaques. They reported that *Pomc* and *Npy* mRNA-containing neurons are found in the ARH of nonhuman primates at gestational day (G) 100. Although only a few AgRP/NPY fibers and no POMC fibers were detected in the PVH at this age, the density of AgRP/NPY fibers innervating the PVH markedly increased at G130 and G170, and POMC fibers were found in the PVH at G170^22^. In human fetuses, NPY-immunoreactive fibers are detected in the ARH and the PVH as early as 21 weeks of gestation^23^.

## Hormonal factors that influence melanocortin neuron development

### Leptin

**Fig. 2:**
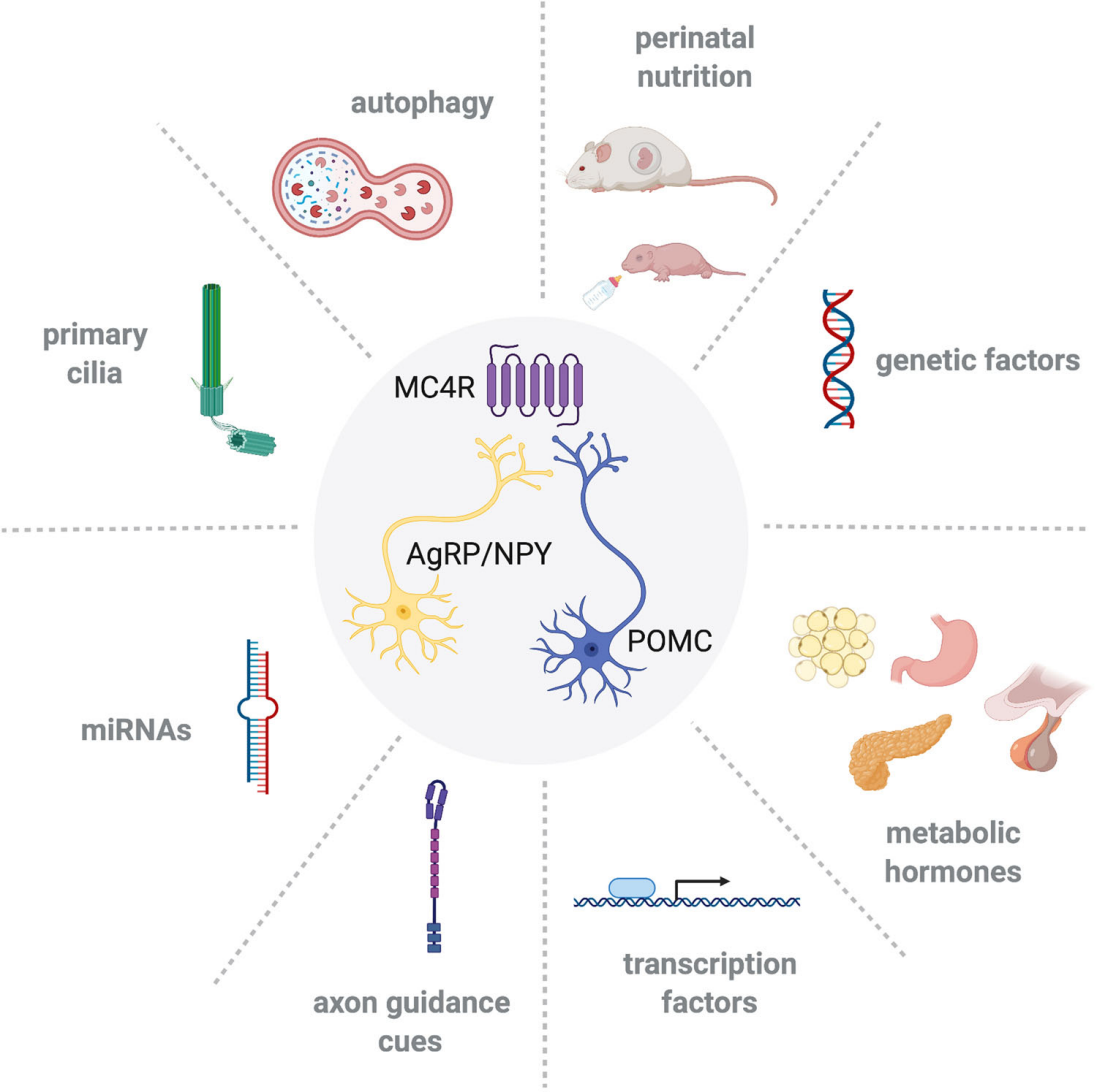
Factors regulating melanocortin system development. Hypothalamic development involves cell-intrinsic cellular and molecular factors as well as endocrine signals and genetic and environmental factors.

The discovery of leptin led to a paradigm shift in understanding how food intake and body weight can be powerfully and dynamically regulated by hormonal signals^24–26^. In 1994, Friedman et al.^24^ used positional cloning and found that the *ob* gene encodes the hormone leptin, which is secreted by adipose tissue in proportion to its mass. Subsequently, other groups reported that leptin administration reduces body weight and food intake in leptin-deficient mice and humans^27–29^. A few years later, Ahima and colleagues reported that circulating leptin levels exhibit a distinct surge between P8 and P12 in mice^30^, yet exogenous leptin does modulate food intake, growth, or energy expenditure at this developmental stage^31–34^. Instead of acutely regulating food intake and body weight, neonatal leptin appears to be an important neurodevelopmental factor that influences the wiring of hypothalamic circuits (Fig. 2). The labeling of ARH axons combined with immunohistochemical analyses showed that POMC and AgRP neuronal projections are disrupted in leptin-deficient (*ob/ob*) mice^35^. The site of action for the developmental effects of leptin includes at least a direct action on ARH neurons because leptin induces neurite extension from isolated organotypic explants of the ARH ex vivo^35^. Remarkably, leptin appears to exert its developmental action on POMC neural projections during a discrete developmental critical period: exogenous leptin treatment up to P28 rescues AgRP projections in *ob/ob* mice^35,36^. In contrast, leptin treatment of *ob/ob* mice after P28 is relatively ineffective because it did not increase the density of either POMC or AgRP fibers in the PVH to levels that are characteristic of wild-type mice^35,36^. Together, these observations suggest the existence of a critical period for the neurotrophic effect of leptin on POMC and AgRP circuits that closes around puberty. More in-depth studies have examined leptin receptor signaling pathways that mediate the axonotrophic effect of leptin. The leptin receptor exists in several alternatively spliced isoforms, of which only the long form (LepRb) associates with Janus kinase 2 to mediate intracellular signaling. LepRb initiates multiple intracellular signal transduction pathways upon leptin binding that result in the activation of STAT family transcription factors, extracellular signal-regulated kinases (ERKs), and phosphoinositol-3 kinase/Akt. Developing POMC and AgRP neurons express LepRb^37^, and leptin administration in mouse neonates results in the activation of major LepRb signaling pathways, including STAT3, ERK, and Akt^37,38^. The disruption of POMC and AgRP axonal projections is observed in mice or rats that lack functional LepRb signaling (*db/db* mice and *fa/fa* rats, respectively)^38,39^. Moreover, a lack of functional LepRb→STAT3 signaling in vivo (*s/s* mice) or in vitro results in a reduced density of POMC fibers without altering the development of AgRP projections, showing the importance of this signaling pathway, specifically, in the development of POMC neural projections^38^. However, not all LepRb signaling pathways play a role in the formation of ARH projections. For example, mice that lack LepRb→ERK signaling (*l/l* mice) display densities of POMC and AgRP axons in the PVH that are comparable to those of wild-type mice^38^.

### Ghrelin and growth hormone

Ghrelin is a 28 amino acid hormone peptide that is mainly produced by the stomach and is an endogenous ligand for the growth hormone secretagogue receptor (GHSR). It is one of the most potent orexigenic signals that exerts its action on food intake by stimulating AgRP/NPY neurons, which in turn inhibit POMC neurons^40,41^. Although the marked orexigenic effect of ghrelin is not yet present before weaning in mice or rats^42,43^, ghrelin in early postnatal life does have a lasting developmental effect on the hypothalamic circuits involved in energy homeostasis^43^ (Fig. 2). Mice injected with an anti-ghrelin compound during neonatal life display increased densities of POMC- and AgRP-containing axons innervating the PVH. These structural alterations are accompanied by long-term metabolic defects, including elevated body weight, increased adiposity, and hyperglycemia^43^. However, if adult mice are treated with the anti-ghrelin compound, it does not alter POMC and AgRP circuits^43^. These findings suggest that, similar to leptin, the developmental action of ghrelin on arcuate projections is restricted to a critical neonatal window. The site of action for the developmental effects of ghrelin likely includes direct action on arcuate neurons because direct exposure of isolated ARH explants to ghrelin inhibits axonal outgrowth^43^. Ghrelin also interacts with LepRb→STAT3 signaling to block the neurotrophic effect of leptin^43^.

Ghrelin is a potent stimulator of growth hormone secretion^44^. Based on the documented finding that ghrelin influences hypothalamic development and can interact with leptin receptor signaling^43^, it is not surprising that the deletion of the growth hormone receptor in *Leprb*-expressing cells also alters the development of POMC and AgRP neuronal circuits^45^. In addition, selective loss of the growth hormone receptor in AgRP neurons affects AgRP axonal projections without affecting POMC circuits^45^, demonstrating a cell-autonomous effect of growth hormone on AgRP neuronal development.

### GLP1

The incretin hormone glucagon-like peptide 1 (GLP1) is secreted postprandially by intestinal enteroendocrine cells to promote satiety and glucose-induced insulin release^46^. The administration of the GLP1-R agonist exendin-4 during the first week of postnatal life decreases the density of NPY fibers innervating the PVH and has a protective effect against both age-related and diet-induced obesity^47^. Moreover, genetic deletion of *Glp1r* in *Sim1* neurons of the PVH reduces AgRP/NPY projections, while it increases POMC projections to the PVH^47^.

### Amylin

Amylin is a hormone produced by pancreatic β-cells and is coreleased with insulin in response to caloric intake. The amylin receptor comprises the core calcitonin receptor (CTR), which heterodimerizes with one or several receptor activity-modifying proteins (RAMP-1, -2, and -3). The primary role of amylin in adults is to reduce food intake by promoting meal-ending satiation and maintaining glucose homeostasis. During development, amylin is detected in the blood circulation of embryos, where it appears to act through RMAP1-3 to influence the neurogenesis of POMC neurons^48^ (Fig. 2). During postnatal life, amylin continues to be secreted in the blood circulation^49^, and a loss of *amylin* or *Ramp1/3* disrupts the development of POMC and AgRP projections to the PVH^50^.

## Molecular programs of melanocortin neuron development

### Transcription factors

Homeobox genes belong to a class of transcription factors that play important roles in regionalization, patterning, and cell differentiation during embryogenesis and organ development. The homeobox genes orthopedia (*Otp*), *Nkx2*.1, and *Bsx* are highly expressed in the ventral hypothalamus during embryonic development. Loss-of-function studies indicated that while *Nkx2.1 and Otp* are essential for the normal morphological development of the hypothalamus, including the ARH, *Bsx* is not required^51–53^ (Fig. 2). The homeobox gene *Nkx2.1*, also known as thyroid transcription factor 1 (*Ttf-1*), plays a particularly important role in ARH specification. The ablation of *Nkx2.1* impairs the formation of the ventral hypothalamic primordium, resulting in the absence of ARH formation^51,54^. However, the ventromedial nucleus (VMH), dorsomedial nucleus (DMH), and lateral hypothalamic area (LHA) are present. The expression of *Nkx2.1* in postmitotic cells suggests that it further plays a role in the differentiation and maintenance of ARH neurons in the ventral portion of the hypothalamus^55,56^. Deficiency in *Nkx2.1* prior to the onset of *Pomc* expression markedly reduces *Pomc* cell numbers^57^. However, the number of NPY neurons was not affected in *Nkx2.1* knockout mice, and the POMC neuronal cell number was not affected if *Nkx2.1* deletion occurred in postmitotic *Pomc* neurons^57^. The LIM-homeodomain transcription factor Islet 1 (*Isl1)* is upregulated in hypothalamic *Nkx2.1* progenitor cells at E10, i.e., just before the onset of neuropeptide expression^58,59^. Consistent with the role of *Isl1* in the phenotypic determination of ARH neurons, loss-of-function studies revealed that *Isl1* promotes the terminal differentiation of *Pomc*, *Agrp*, and *Npy* expression^58,59^. In contrast, the deletion of the transcription factor *Dlx1/2* in *Nkx2.1-expressing progenitors* increases *Agrp* expression without affecting *Pomc* expression^60^. More in-depth molecular studies revealed that *Dlx1/2* controls *Agrp* expression by binding to and repressing the expression of the homeodomain transcription factor *Otp*, which is also known to influence ARH morphogenesis^52,53,60^. Thus, the loss of *Otp* in *Agrp* neurons results in a dramatic reduction in the number of *Agrp* mRNA-expressing cells^61^. Notably, *Otp* expression is absent in the hypothalamus of *Isl1* knockout embryos^58^, suggesting that, in addition to *Dlx1/2*, *Isl1* is also required for the expression of *Otp* in the future ARH. Sonic hedgehog (*Shh*), SIX homeobox 3 (*Six3*), and retinal anterior neural fold homeobox (*Rax*) were also identified as critical regulators of ventral hypothalamus development and POMC development. *Shh* signaling increases *Nkx2.1* expression^62^, and the deletion of *Shh* in *Nkx2.1* progenitors affects the development of POMC neurons^63^. *Six3* is a regulator of forebrain development, including the hypothalamus^64^, and is required for *Shh* expression^65^. Finally, *Rax* is also important for the formation of the ventral neural tube. Mice lacking *Rax* in *Six3*-expressing cells do not show ventral hypothalamic *Nkx2.1* expression and never express POMC^66^.

The oligodendrocyte transcription factor family (*Olig1* and *Olig2*) are basic helix–loop–helix (bHLH) transcription factors highly expressed in the periventricular regions of the brain, such as the hypothalamus^67,68^. Lineage tracing experiments indicate that a number of POMC and NPY cells are derived from *Olig1* progenitors^69^. The majority of *Olig1* progenitors also express bone morphogenetic protein receptor 1A (Bmpr1A)^69^, and when Bmpr1A is deleted in *Olig1*-expressing cells, it decreases and increases the number of POMC and AgRP neurons, respectively^69^. Neurogenin 3 (*Ngn3)* is another bHLH transcription factor expressed in hypothalamic progenitors, and it plays an opposite role in the specification of *Pomc* and *Npy* neurons: while neurogenin 3 promotes the embryonic development of *Pomc* neurons, it inhibits *Npy* neuronal development^70,71^. However, not all POMC neurons are derived from *Ngn3* progenitors. Using a mouse model of *Mash1* deficiency, McNay et al.^72^ further reported that this bHLH transcription factor has a pro-neural function and acts upstream of *Ngn3* to regulate neurogenesis in the ventral hypothalamus. A loss of *Mash1* results in the disappearance of *Ngn3* expression in ARH progenitors and is associated with a dramatic reduction in the number of POMC and NPY neurons^72^. The Notch signaling pathway also appears to mediate its developmental effects on POMC and NPY neuronal development through *Mash1*. Mice lacking Notch signaling in *Nkx2.1*-expressing cells display an increased number of POMC and NPY neurons associated with an induction in *Mash1* expression^72,73^. In addition, mice with a constitutively active *Notch1* intracellular domain show a complete loss of POMC and NPY neurons^73^, mirroring the effects of *Mash1-*deficient mice^72^.

As described above, *Pomc*-expressing progenitors in the ARH have the unique ability to differentiate into functional mature NPY neurons^15^. Recent data from our laboratory investigated the molecular mechanisms involved in this developmental switch and identified miR-103/107 as candidates involved in *Pomc* progenitor differentiation (Fig. 2). A loss of the microRNA (miRNA)-processing enzyme *Dicer* increases the proportion of *Pomc* progenitors acquiring an NPY phenotype^74^. Moreover, the silencing of miR-103/107 specifically decreases the number of *Pomc*-expressing cells and increases the proportion of *Pomc* progenitors differentiating into NPY neurons^74^. Postnatal maintenance of *Pomc* and *Npy* peptidergic identity also depends on the expression of the transcription factor T-box 3 (*Tbx3*)^75^. Because the majority of miRNAs exert their effects on gene expression by targeting transcription factors, it would be interesting to study whether there is a link between miRNAs and *Tbx3* and *Pomc*/*Npy* gene expression.

### Axon guidance molecules

Axons grow by sending out a highly plastic and sensitive structure called a “growth cone,” which travels toward the target with the elongating neurite trailing behind. As described above, metabolic hormones, including leptin, are critical factors influencing initial POMC and AgRP/NPY axon outgrowth. Growing POMC axons must then choose a path to follow and decide the direction to go on this path to innervate the proper nucleus (e.g., the PVH). The pathways are defined by cell–cell interactions and diffusible chemorepulsive and chemoattractive cues^76^. The diffusible axon guidance cues semaphorins are highly expressed in the PVH during development, and POMC neurons express the semaphorin receptors neuropilin 1 and 2 (ref. ^77^). Supporting a role for neuropilins/semaphorins in POMC axon guidance, a loss of neuropilin 2 receptors in POMC neurons specifically disrupts the development of POMC axonal projections to the PVH^77^. These structural alterations are accompanied by metabolic dysregulation, including increased body weight and glucose intolerance^77^. Notably, exome sequencing experiments identified variants in the semaphorin and neuropilin families associated with severe obesity in humans^77^, demonstrating the translational importance of these findings. The formation of POMC neural neuronal connectivity also involves cell–cell adhesion proteins. Supporting this idea, POMC neurons are enriched in the cell-to-cell contact molecules *Efnb1* (EphrinB1) and *Efnb2* (EphrinB2) during postnatal development, and a loss of *Efnb1* or *Efnb2* in *Pomc*-expressing progenitors decreases the amount of excitatory glutamatergic inputs^78^. In addition, mice that are deficient in *contactin*, a cell adhesion molecule involved in the formation of axonal projections, have a reduced density of POMC fibers in the PVH during postnatal development^79^.

## Cellular factors underlying melanocortin neuron development

### Autophagy

Axonal growth involves dynamic remodeling of cytosolic structures and requires protein degradation and turnover to replace damaged organelles and proteins. The maintenance of cell function and growth is achieved with autophagy, which is an important cellular degradation system that engulfs parts of the cytoplasm and organelles within double-membrane vesicles, known as autophagosomes, to turn over and recycle these cellular constituents^80^. This cellular process is also critical in the supply of nutrients for survival during starvation^81^. Constitutive autophagy is detected in the hypothalamus, including in arcuate POMC neurons, during critical periods of axon growth and development^82^. A loss of the autophagy-related protein (*Atg*) gene *Atg7* disrupts the maturation of POMC axonal projections and causes lifelong metabolic perturbations^82^ (Fig. 2). As described above, leptin is a critical neurotrophic factor for POMC circuits, and direct crosstalk has been described between leptin and hypothalamic autophagy during perinatal life^83^. Supporting a role for autophagy in mediating the trophic effects of leptin, a loss of autophagy in POMC neurons exacerbates the metabolic and neurodevelopmental deficits observed in leptin-deficient *ob/ob* mice^83^.

### Primary cilia

Another cellular signaling system that plays an important role in brain development and function is the primary cilium, which is a microscopic sensory antennae that cells in many vertebrate tissues use to gather information about their environment. It is an organelle found on the cell surface of most mammalian cells, including hypothalamic neurons^84^. For example, during embryonic development, primary cilia are important mediators of *Shh*, which is a critical regulator of the ventral patterning of the hypothalamus^85^. Moreover, a strong interaction between primary cilia and autophagy has been reported, including in the developing hypothalamus^86^. Cilia begin to be observed in hypothalamic neurons on E12, and the number and length of primary cilia gradually increase thereafter to reach an adult-like pattern at P14 (ref. ^86^). A disruption of cilia formation in developing POMC neurons, but not in adult POMC neurons, increases body weight, fat mass, and food intake; reduces energy expenditure; and alters glucose homeostasis during adult life^86^. Neuroanatomically, a reduction in the number of POMC neurons was observed in juvenile mice lacking primary cilia on POMC neurons, but POMC cell numbers were normal in adult mutant mice. This reduction in POMC cell numbers during the preweaning period is attributed to a decrease in neurogenesis during embryonic development (in opposition to an effect on apoptosis), and the adult normalization in POMC cell number is attributed to a compensatory increase in neurogenesis after weaning^86^. A loss of primary cilia also alters axonal and dendritic growth, resulting in a reduced density of POMC fibers innervating the PVH, DMH, and LHA^86^ (Fig. 2). It also blocks the ability of leptin to promote the development of POMC projections in *ob/ob* mice^86^. Because a subpopulation of *Pomc* progenitor cells also give birth to NPY neurons (see above and ref. ^15^), it is not surprising that mice lacking primary cilia from embryonic POMC neurons also display a reduction in NPY cell numbers and NPY fibers innervating the PVH^86^. The interaction between primary cilia and the melanocortin system appears bidirectional. Consistent with this idea, Siljee et al.^87^ reported that MC4-R colocalizes with adenylate cyclase 3 in the primary cilia of a subset of PVH neurons and that the inhibition of primary cilia adenylyl cyclase signaling increases body weight.

## Effect of perinatal nutrition on the development of the melanocortin system

Obesity has reached alarming rates worldwide and is associated with several life-threatening conditions, such as hypertension and type-2 diabetes. The prevalence of obesity among pregnant women is also at an all-time high, and it represents a significant risk factor for the development of metabolic diseases in offspring. Obesity is determined by genetics and obesogenic environments, such as diets rich in fat and/or sugar. Maternal high-fat diet (HFD) feeding during pregnancy and/or lactation in rodents is a useful experimental approach for studying the consequences of maternal obesity on the development of offspring’s future health outcomes. Similar to what is observed in humans, offspring born to obese females fed an HFD (45–60% of calories from fat) during gestation and/or lactation become progressively overweight, hyperphagic, and glucose intolerant, and they display an increase in adiposity^88,89^. These metabolic alterations are associated with a disrupted development of POMC and AgRP/NPY projections to the PVH in both mice and rats^89–93^ (Fig. 2). Notably, maternal consumption of an HFD during lactation alone (but not during pregnancy alone) appears sufficient to cause obesity and diabetes and alter the development of POMC and AgRP projections^90^, showing the importance of postnatal nutrition, specifically, in hypothalamic programming. A similar reduction in AgRP axonal projections has been reported in nonhuman primates following maternal HFD exposure^91^. The rat model of diet-induced obesity (DIO) developed by Levin et al.^94^ also provides a valuable tool for studying obesity, in part because Levin’s DIO rats share several features with humans with obesity, including polygenic inheritance. Therefore, this animal model is particularly well suited for studying the relative contribution of genetic versus environmental factors in metabolic programming. Animals born to genetically obese-prone DIO dams display a reduction in the number of POMC and AgRP axons innervating the PVH^95^. In addition, significant remodeling of synapses onto POMC neurons has been observed in DIO rats, particularly in response to nutritional challenges^96^. DIO rats fed a chow diet display increased inhibitory inputs to POMC neurons compared to obesity-resistant rats. In addition, DIO rats fed an HFD display a loss of synapses onto POMC neurons, whereas high-fat feeding in control obesity-resistant rats causes an increase in POMC synaptic coverage^96^.

The precise mechanisms that underlie the maternal obesity-induced alterations in melanocortin system development have only begun to be elucidated. Several studies have indicated that abnormal leptin and insulin signaling during postnatal development may represent a likely cause of HFD- and DIO-induced alterations in hypothalamic development. For example, DIO rats and animals born to obese dams display an abnormal organization of projection pathways derived from the ARH that appear to be the result of the diminished responsiveness of ARH neurons to the trophic actions of leptin during critical periods of postnatal development^95^. Moreover, animals born to obese dams are hyperleptinemic, which is associated with hypothalamic leptin resistance, and improving leptin sensitivity with the endoplasmic reticulum stress-relieving drug tauroursodeoxycholic acid normalizes metabolic and neurodevelopmental deficits in these animals^91^. At the cellular level, a reduction in cilia length and frequency has been reported in the ARH of pups born to obese dams, suggesting that the alteration of this cellular system is critical for hypothalamic development and that leptin signaling could contribute to obesity-induced perturbations of hypothalamic development^86^. Changes in insulin signaling could also mediate the neurodevelopmental effects of maternal obesity. Mothers fed an HFD and their offspring are hyperinsulinemic, and deleting the insulin receptor in POMC neurons prevents the diet-induced disruption of POMC projections^90^. More recently, Dearden et al.^97^ reported that the offspring of obese dams display a reduction in POMC cell numbers that likely results from a diminished neurogenic action of insulin during embryonic development. Similarly, amylin, which is coreleased with insulin by pancreatic β-cells, appears to be involved in the nutritional programming of melanocortin circuits. The offspring of obese dams are hyperamylinemic from embryonic age throughout adulthood. Amylin fails to activate hypothalamic amylin receptor signaling if animals are born to obese mothers, which is associated with an inability of amylin to promote POMC neurogenesis^48^. Similarly, knocking down CTR in the ventromedial part of the hypothalamus of obesity-resistant rats (a region that encompasses the ARH + VMH) alters the development of POMC circuits^98^. In contrast, neonatal amylin treatment in DIO rats enhances STAT3 signaling in the ARH, accompanied by a restoration of AgRP and POMC fibers^98^.

In part because of the importance of postnatal brain development in rodents, including that of POMC and AgRP neurons, animal models of postnatal metabolic programming have also been extensively studied. The reduction in litter size in rodents has proven to be a very useful model to manipulate the postnatal diet specifically. This model was first characterized by the pioneering works of Kennedy^99^, Widdowson and McCance^100^, who reported more than 60 years ago that pups raised in small litters (SL) display an almost fourfold difference in preweaning growth compared to pups raised in large litters. This weight difference persisted throughout life and even increased as the rats were weaned onto a regular chow diet. Similar to animals born to obese dams, pups raised in SL display early life hyperleptinemia associated with hypothalamic leptin resistance^101,102^. Postnatal overnutrition also results in reduced responsiveness of hypothalamic neurons to ghrelin during neonatal life, which appears to be the consequence of altered transport of the hormone across the blood–brain barrier^103^. These endocrine changes during critical periods of postnatal development are associated with a dysregulation of the melanocortin system (Fig. 2). Reduced arcuate *Pomc* expression has been reported in rats raised in SL^87,104^. It is known that epigenetic mechanisms of gene regulation, such as DNA methylation and histone modifications, such as methylation, regulate gene expression in response to environmental stimuli. Plagemann and colleagues therefore studied the methylation status of CpG dinucleotides of the *Pomc* promoter in rats raised in SL. They found that postnatal overfeeding causes alterations in the methylation of the *Pomc* promoter, particularly on the CpG dinucleotides within the two *Sp1*‐related binding sequences (Sp1, NF‐κB) that are critical for leptin and insulin’s effects on *Pomc* gene expression^105^. Similarly, human studies have shown that childhood obesity is associated with *Pomc* hypermethylation^106^. In addition to altering *Pomc* gene expression in the ARH, postnatal overfeeding also disrupts the neurophysiological responses of PVH neurons to αMSH and AgRP^107^.

## Conclusion

It is now clear from a variety of epidemiological and experimental studies that many chronic diseases, including obesity and type-2 diabetes, may have their roots during neonatal development. A perturbed environment in early life is thought to elicit a range of cellular adaptive responses in key systems, the melanocortin system being one of them. In rodents, the development of the melanocortin system is initiated during mid-gestation and continues during the postnatal period. These developmental windows represent important periods of vulnerability during which perturbations in the perinatal environment may lead to abnormal POMC and AgRP neuron development, causing lifelong metabolic diseases. Therefore, it will be critical to have a comprehensive knowledge of factors that are detrimental or beneficial for the development of the melanocortin system if we want to design intervention studies or treatments. The marked species difference in terms of hypothalamic developmental trajectories is important to consider, as rodents and humans may exhibit different periods of vulnerability to developmental insults or different responses to therapeutic interventions based on the temporal and regional maturation patterns of the hypothalamus. Nevertheless, the mature hypothalamus, whether in rodents or primates, can still exhibit neuroplastic responses, although the degree and nature of hypothalamic remodeling may differ between adults and neonates.
